# Ancylostomatidae in wild canids and felids from Romania: new host associations and haplotype diversity

**DOI:** 10.1186/s13071-025-07219-7

**Published:** 2026-02-04

**Authors:** Ioana Bianca Mitrea, Andreea Daniela Iani, Călin Mircea Gherman, Cristina Daniela Cazan, Angela Monica Ionică, Ștefan Ovidiu Rabei, Georgiana Deak, Mihai Sorin Cernea, Vasile Alexe, Gabriel Bogdan Chișamera, Mihai Marinov, Andrei Daniel Mihalca

**Affiliations:** 1 Department of Parasitology and Parasitic Diseases, Faculty of Veterinary Medicine, University of Agricultural Sciences and Veterinary Medicine of Cluj-Napoca, Calea Mănăștur 3‑5, 400372 Cluj-Napoca, Romania; 2 Department of Pharmacology, Faculty of Veterinary Medicine, University of Agricultural Sciences and Veterinary Medicine of Cluj-Napoca, Calea Mănăștur 3‑5, 400372 Cluj-Napoca, Romania; 3 Ross University School of Veterinary Medicine, Basseterre, Saint Kitts and Nevis; 4 Clinical Hospital of Infectious Diseases of Cluj-Napoca, Iuliu Moldovan 23, 400348 Cluj-Napoca, Romania; 5 Danube Delta National Institute for Research and Development, 820112 Tulcea, Romania; 6 Institute of Biology-Bucharest, Romanian Academy, Splaiul Independenței 296, 060031 Bucharest, Romania; 7 Parasitology Consultancy Group, Corușu 145B, 407056 Corușu, Romania

**Keywords:** Hookworms, Wild carnivores, *Ancylostoma*, *Uncinaria*

## Abstract

**Background:**

Hookworms (Ancylostomatidae) significantly impact on the health of both domestic animals and humans worldwide, with some species capable of causing zoonotic diseases. While hookworm infections in pets are frequently reported in Europe primarily through coproscopic studies, there are limited data regarding their presence in wild carnivores. To address this, this study aimed to assess the diversity, prevalence, and distribution of hookworms in wild canids and felids from Romania through both morphological and molecular analyses.

**Methods:**

From November 2011 to February 2025, 319 carcasses belonging to six species of wild canids and felids from Romania [23 gray wolves (*Canis lupus*), 137 golden jackals (*Canis aureus*), 79 red foxes (*Vulpes vulpes*), 2 raccoon dogs (*Nyctereutes procyonoides)*, 70 European wildcats (*Felis silvestris*), and 8 Eurasian lynxes (*Lynx lynx*)] were collected as road kills or legally hunted. Hookworms were recovered from the intestinal tract during necropsy and preserved in formalin for morphological examination and in absolute ethanol for genetic analysis. Genomic DNA was extracted and analyzed using a PCR targeting a barcode region of the second nuclear ribosomal internal transcribed spacer (ITS-2), followed by sequencing. Sequencing results were compared with other entries from GenBank™.

**Results:**

The overall hookworm infection rate was 14.1%, with hookworms detected in 4 wolves (17.4%), 23 golden jackals (16.8%), 11 European wildcats (15.7%), 4 red foxes (5.1%), 2 raccoon dogs (100%), and 1 lynx (12.5%). Three hookworm species were identified: *Uncinaria stenocephala*, *Ancylostoma caninum*, and *A. tubaeforme*. Molecular analysis revealed 14 unique sequences, comprising nine haplotypes of *U. stenocephala*, three of *A. caninum*, and two of *A. tubaeforme*. We report for the first time the Eurasian lynx as a host for *A. caninum*, expanding the known host range of this species.

**Conclusions:**

This study provides the first comprehensive molecular assessment of hookworm diversity in European wild carnivores, showing new host–parasite associations and highlighting the importance of these hosts as reservoirs for domestic pets and, potentially, humans. The detected haplotypes showed high similarity to isolates from Europe, Asia, and the Americas, indicating a broad global connectivity of hookworm populations.

**Graphical abstract:**

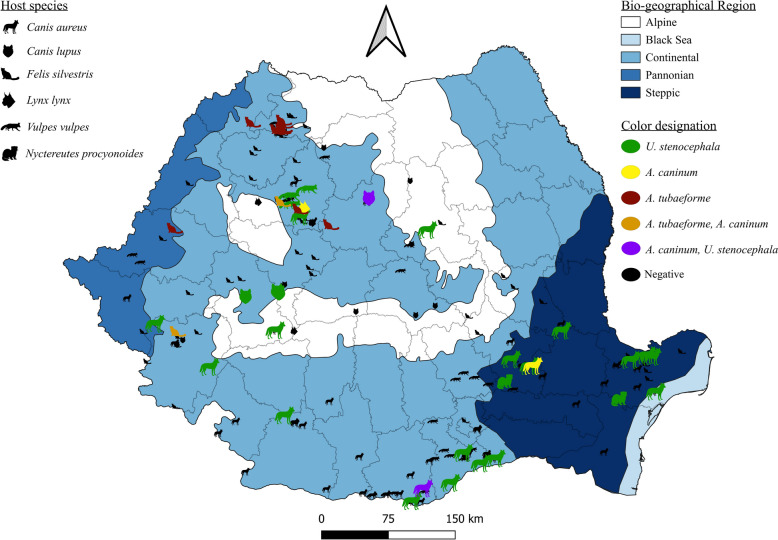

**Supplementary Information:**

The online version contains supplementary material available at 10.1186/s13071-025-07219-7.

## Background

Parasites have a significant negative effect on human and animal health worldwide. Wildlife species shares many parasites with domestic animals, particularly in closely related taxa such as domestic pets and wild carnivores (i.e., dogs versus wild canids; domestic cats versus wild felids). Several of these pathogens can spill over between wildlife and domestic animals and vice versa, the latter situation sometimes posing a threat to the conservation efforts of endangered species [1].

Helminths are widespread parasites infecting wild and domestic carnivores worldwide [2]. Gastrointestinal nematodes of the family Ancylostomatidae (commonly known as hookworms), particularly species of the genera *Ancylostoma* and *Uncinaria*, are among the most pathogenic zoonotic nematodes of domestic carnivores. In addition, they might occasionally impact the health of wild carnivores, causing anemia or other conditions [3].

These hematophagous intestinal parasitic nematodes can seriously impact the health of both humans and animals, their pathogenicity being related to blood loss, which can result in anemia and, in extreme cases, death [4]. They are clinically relevant in dogs, cats, ruminants, pigs, primates, and other hosts [5, 6]. The life cycle of hookworms is typically direct, with females producing thin-shelled eggs that are excreted in the host’s feces. Soil contaminated with L3 resulting from eggs is the primary source of infection. Prevalence rates may vary in relation to climatic regions [7].

Among species that are parasitic in carnivores, *Ancylostoma* spp. are predominantly found in warm and humid climatic zones, mostly in Asia and other tropical countries [8], while *Uncinaria stenocephala* is more common in temperate and subarctic regions [2]. In Europe, the most common hookworms found in domestic carnivores are *A. caninum*, *A. tubaeforme*, and *U. stenocephala*, while among wild carnivores, a wide range of hosts, such as red foxes (*Vulpes vulpes*), wolves (*Canis lupus*), jackals (*Canis aureus*), and wildcats (*Felis silvestris*) are also infected by these parasites [8].

Species of *Ancylostoma* are the most common zoonotic hookworms transmitted from animals to humans, posing a major public health concern, especially in resource-limited regions. Carnivores, as the primary definitive hosts, contribute to the spread of these parasites by contaminating the environment with infective eggs and larvae. Human infections typically occur through ingestion of larvae or skin penetration, leading to conditions such as cutaneous larva migrans, eosinophilic enteritis, and follicular dermatitis [8]. Given their wide distribution and the close interaction between humans, animals, and shared environments, these parasites pose a significant One Health challenge.

In Romania, research on hookworm diversity and epidemiology in wild carnivores is limited, with few reports in red foxes [9, 10], gray wolves, and European wildcats [9, 11]. In domestic carnivores, most studies are based on the identification of eggs in feces [12–18]. However, the specific identification on the basis of egg detection in feces remains doubtful owing to the overlap of egg size and the presence of co-infections [8, 19].

Moreover, surveys on hookworms in wild carnivores in Europe are also scarce and limited to a few countries and hosts. In this context, this study aimed to assess the diversity and host spectrum of hookworms in wild carnivores from Romania by combining morphological and molecular methods.

## Methods

### Sample collection

**Fig. 1:**
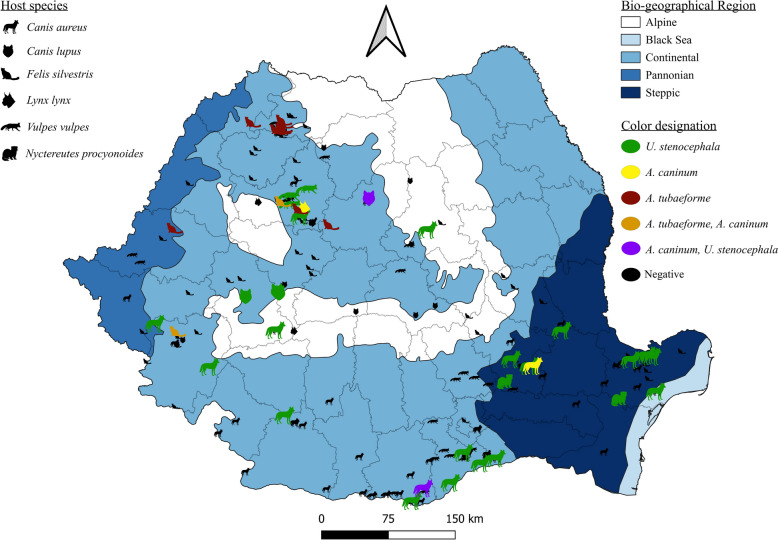
Distribution of collected samples. Maps were created using QGis v3.40.11 software (2025; www.qgis.org/en/site) with the following layers: Biogeographical regions 2016 (www.eea.europa.eu) and Romania shapefile (www.geo-spatial.org)

Between November 2011 and February 2025, carcasses of 23 gray wolves (*Canis lupus*), 137 golden jackals (*Canis aureus*), 79 red foxes (*Vulpes vulpes*), 2 raccoon dogs (*Nyctereutes procyonoides*), 70 wildcats (*Felis silvestris*), and 8 Eurasian lynxes (*Lynx lynx*), were collected as road kills or legally hunted from different locations from Romania and examined at the Department of Parasitology and Parasitic Disease of the University of Agricultural Sciences and Veterinary Medicine of Cluj-Napoca (Dataset 1; Fig. 1). All the carcasses were properly labeled and kept at −20 °C until further examination.

### Postmortem investigation of gastrointestinal nematodes

A complete parasitological necropsy [20] was performed on all carcasses. To collect the hookworms, the digestive tract was removed and opened longitudinally. The intestinal mucosa was scraped with the blunt side of a knife blade and placed into sedimentation cones, washed, and examined under the stereomicroscope in a Petri dish. Adult hookworms were collected, washed in saline solution, and preserved in tubes with absolute ethanol for molecular analyses and in 10% formalin for morphological examination.

### Morphological identification

In total, 260 hookworms from 30 animals (11 females and 13 males from gray wolves, 130 females and 64 males from golden jackals, 16 females and 14 males from European wildcats, 8 females and 3 males from red foxes, and 1 female from a raccoon dog), randomly selected, were examined under an Olympus BX-61 microscope and morphologically identified to species level using available keys and descriptions [4, 21–24].

### Molecular confirmation

Molecular analyses were carried out on 48 adult nematodes (3 females and 4 males from gray wolves, 10 females and 8 males from golden jackals, 10 females and 7 males from European wildcats, 2 females and 2 males from red foxes, 1 female from a raccoon dog, and 1 female from a Eurasian lynx) preserved in absolute ethanol from each host. Genomic DNA was extracted using the ISOLATE II Genomic DNA Kit (Meridian Bioscience, London, UK), following the manufacturer’s protocol, and stored at −20 °C. DNA samples were screened by conventional PCR targeting the second internal transcribed spacer (ITS-2) of the ribosomal DNA of the nematodes, selected for its level of genetic variation that allows reliable species differentiation, by using the previously described primers NC1 (5′-ACG TCT GGT TCA GGGTTG TT-3′) and NC2 (5′-TTA GTT TCT TTT CCT CCG CT-3′) [25]. Each 25 μl PCR reaction contained 12.5 μl Green PCR Mastermix (Rovalab GmbH, Teltow, Germany), 6.5 μl of ultrapure water, 1 μl (10 pmol/μl) of each of the two previously mentioned primers, and a 4 μl aliquot of isolated DNA. Amplifications were performed in a C1000™ Thermal Cycler (Bio-Rad, London, UK) with the following conditions: initial denaturation at 95 °C for 5 min, followed by 40 cycles of denaturation at 95 °C for 45 s, annealing at 60 °C for 45 s, and extension at 72 °C for 45 s, with a final extension at 72 °C for 5 min. PCR products were separated via electrophoresis on a 1.5% agarose gel stained with ECO Safe 20,000× Nucleic Acid Staining Solution (Pacific Image Electronics, New Taipei, Taiwan).

Band sizes were estimated by comparison with a 100 bp HyperLadder (Meridian Bioscience, London, UK). Subsequently, the amplified products were then purified using the Gel/PCR DNA Fragments Kit (Geneaid Biotech, New Taipei, Taiwan) and submitted for sequencing at Macrogen Europe (Amsterdam, the Netherlands). The obtained sequences were assembled and edited using geneious^®^ software (Biomatters LTD., Auckland, New Zealand) and compared with existing sequences from GenBank^®^ by means of Basic Local Alignment Search Tool (BLAST) analysis.

The phylogenetic analysis was performed using MEGA X software [26]. The sequences obtained during the present study were aligned with other sequences downloaded from GenBank using the MUSCLE algorithm, and evolutionary history was inferred by using the maximum likelihood method and Tamura 3-parameter model [27].

## Results

**Fig. 2:**
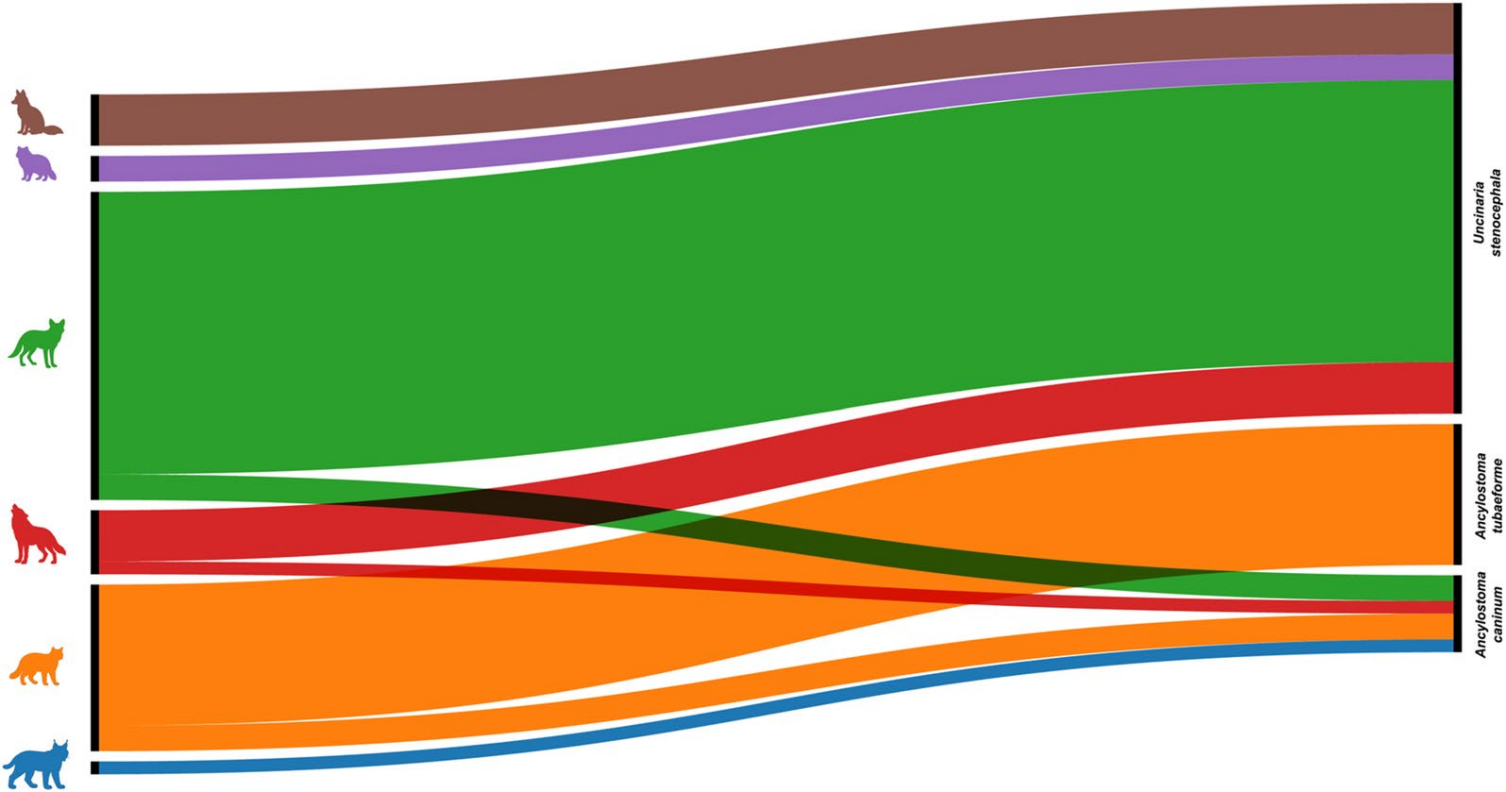
Host–parasite associations between hookworms and wild canids and felids in Romania. The thickness of each line is proportional to the number of positive cases (brown: red fox, purple: raccoon dog, green: golden jackal, red: gray wolf, orange: European wildcat, and blue: Eurasian lynx)

**Fig. 3:**
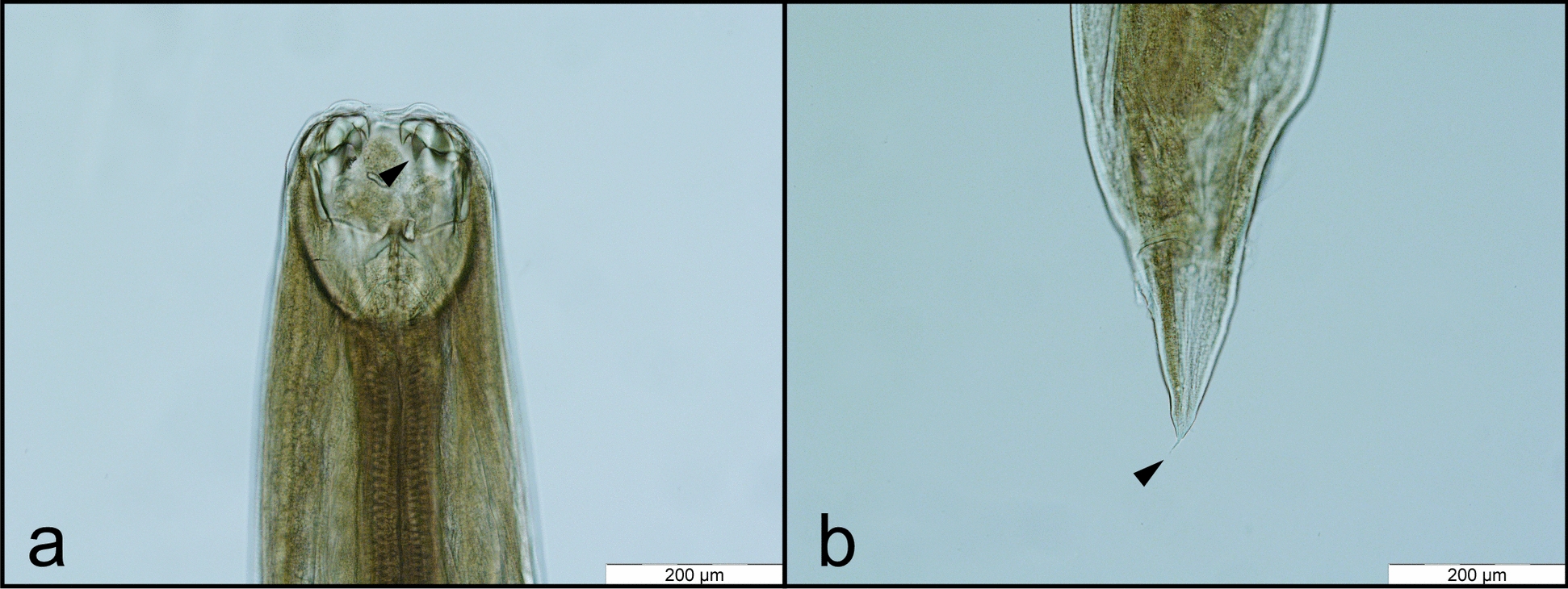
*A. caninum*: cephalic extremity with the oral opening armed with nearly equally sized teeth (arrowhead), dorsal view (**a**). Female tail, ending with the terminal spine (arrowhead), lateral view (**b**)

**Fig. 4:**
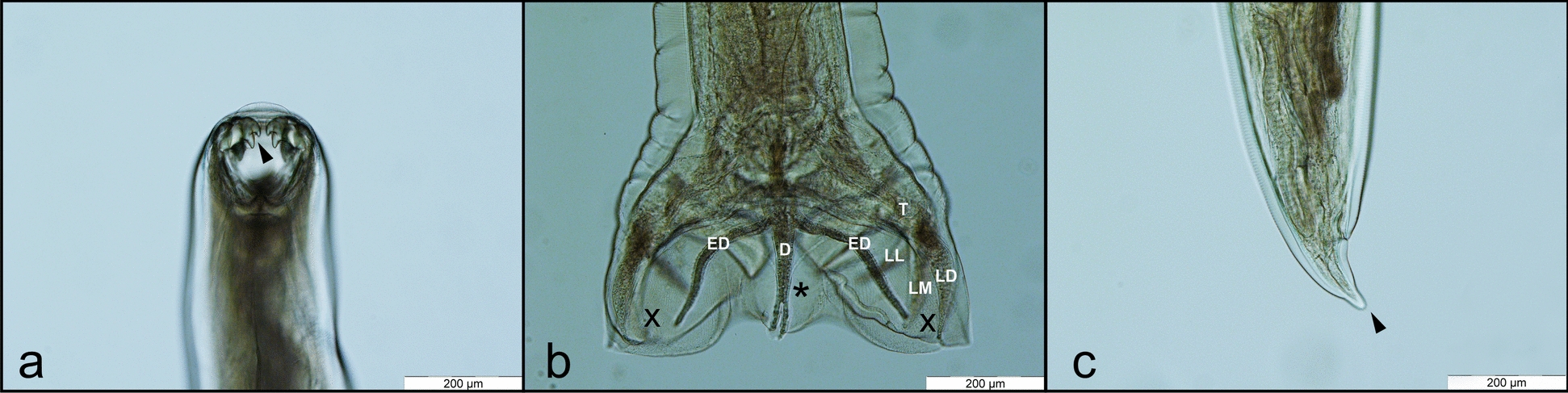
*A. tubaeforme*: cephalic extremity with the oral opening armed with unequal teeth (black arrowhead), with the lateral ones being significantly larger; dorsal view (**a**). Male posterior extremity, with the copulatory bursa; dorsal view (**b**). The male bursa presents three lobes, of which the medial (*) is small and the lateral lobes (X) are well developed. Originating from a single trunk (T), the latero-dorsal ray (LD), latero-medial ray (LM), and latero-lateral ray (LL) diverge from one another. The dorsal ray (D) splits distally into two equal branches. The externo-dorsal ray (ED) arising from dorsal ray; female tail, ending with the terminal spine (black arrowhead); lateral view (**c**)

**Fig. 5:**
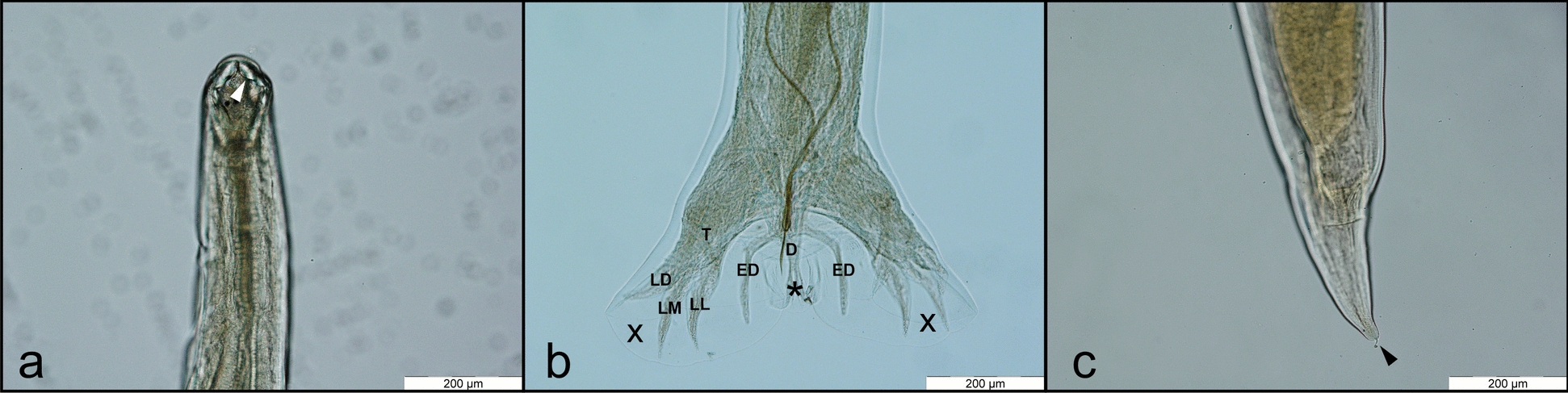
*U. stenocephala*: cephalic extremity with the oral opening armed with cutting plates (white arrowhead), dorsal view (**a**). Male posterior extremity, with the copulatory bursa; dorsal view (**b**). The male bursa presents three lobes, of which the medial (*) is small and the lateral lobes (X) are well developed. Originating from a single trunk (T), the latero-dorsal ray (LD), latero-medial ray (LM), and latero-lateral ray (LL) diverge from one another. The dorsal ray (D) splits distally into two equal branches. The externo-dorsal ray (ED) arising from dorsal ray; female tail, ended with the terminal spine (black arrowhead); lateral view (**c**)

From all the carcasses examined, hookworm infection was detected in 4 Gy wolves, 23 golden jackals, 4 red foxes, 2 raccoon dogs, 11 European wildcats, and 1 Eurasian lynx (Table 1; Fig. 2). Overall, three hookworm species were identified: *A*. *caninum* (Fig. 3) in gray wolf, golden jackal, European wildcat, and Eurasian lynx, *A*. *tubaeforme* (Fig. 4) in European wildcat, and *U*. *stenocephala* (Fig. 5) in gray wolf, golden jackal, red fox, raccoon dog, and European wildcat.

**Table 1:** Prevalence of hookworm infection in wild canids and felids from Romania

Host	Examined	Positive (%)	AC (%)	AT (%)	US (%)	AC + US (%)	AC + AT (%)
Gray wolf	23	4 (17.4)	−	−	3 (13.0)	1 (4.3)	−
Golden jackal	137	23 (16.8)	1 (0.7)	−	21 (15.3)	1 (0.7)	−
Red fox	79	4 (5.1)	−	−	4 (5.1)	−	−
Raccoon dog	2	2 (100)	−	−	2 (100)	−	−
Total canids	241	33 (13.7)	1 (0.4)	−	30 (12.4)	2 (0.8)	−
European wildcat	70	11 (15.7)	−	9 (12.9)	−	−	2 (2.9)
Eurasian lynx	8	1 (12.5)	1 (12.5)	−	−	−	−
Total felids	78	12 (15.3)	1 (1.2)	9 (11.5)	−	−	2 (2.5)
Total	319	45 (14.1)	2 (0.6)	9 (2.8)	30 (9.4)	2 (0.6)	2 (0.6)

AC, *A. caninum*; AT, *A. tubaeforme*; US, *U. stenocephala*; AC + US, co-infection of *A. caninum* + *U. stenocephala*; AC + AT, co-infection of *A. caninum* + *A. tubaeforme*

**Fig. 6:**
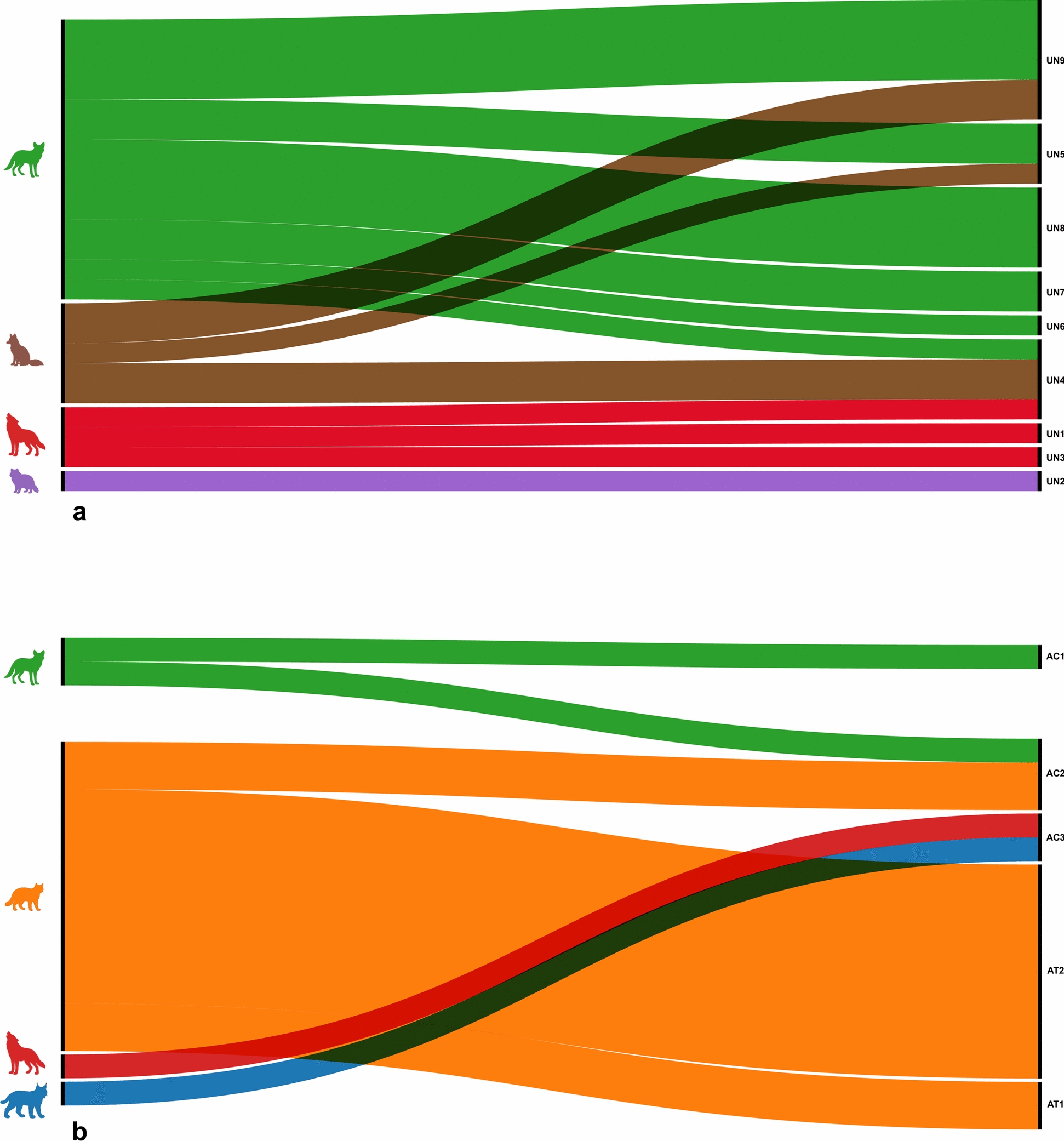
Host–haplotype associations for hookworm species detected in wild carnivores from Romania. The thickness of each line is proportional to the number of hosts carrying a given haplotype. **a** Host–haplotype associations for *U. stenocephala* (UN1–UN9). **b** Host–haplotype associations for *A. caninum* (AC1–AC3) and *A. tubaeforme* (AT1–AT2) (green: golden jackal, brown: red fox, red: gray wolf, purple: raccoon dog, orange: European wildcat, and blue: Eurasian lynx)

**Fig. 7:**
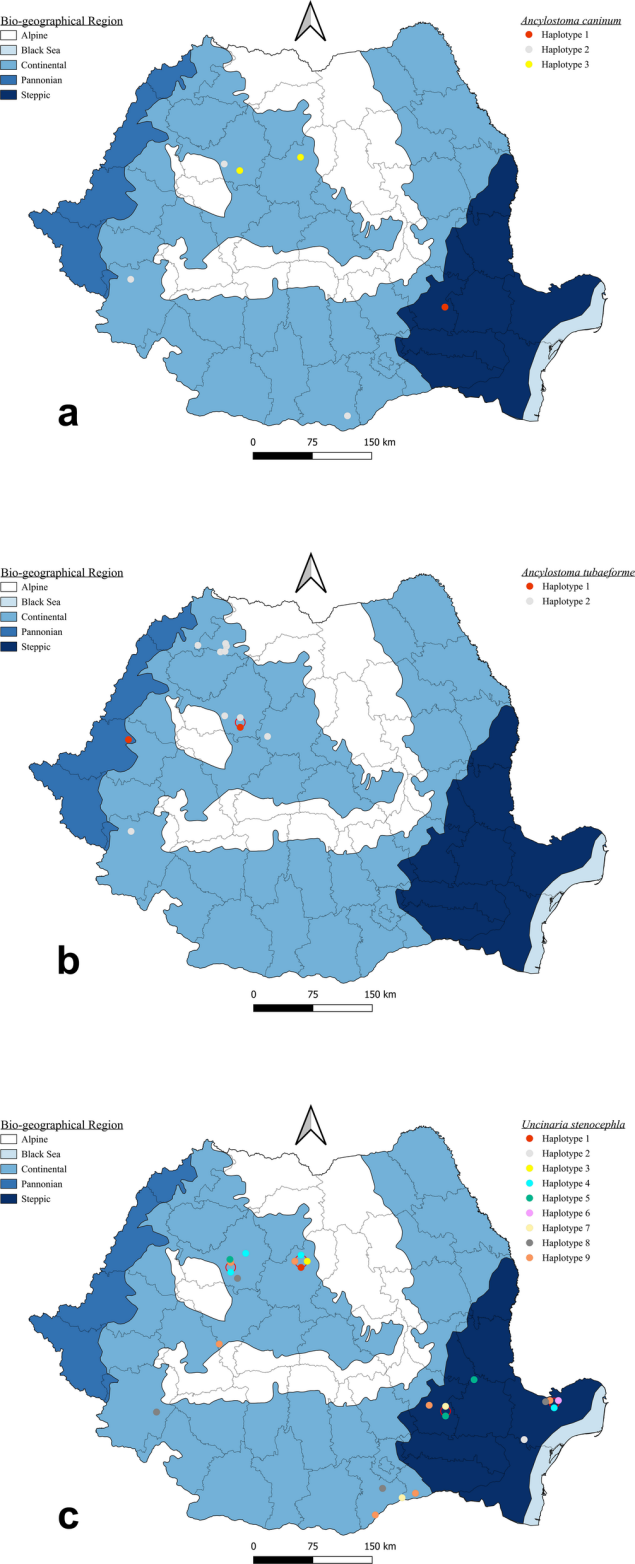
Geographical distribution of haplotypes. **a** *Ancylostoma caninum*. **b** *Ancylostoma tubaeforme*. **c** *Uncinaria stenocephala*

**Fig. 8:**
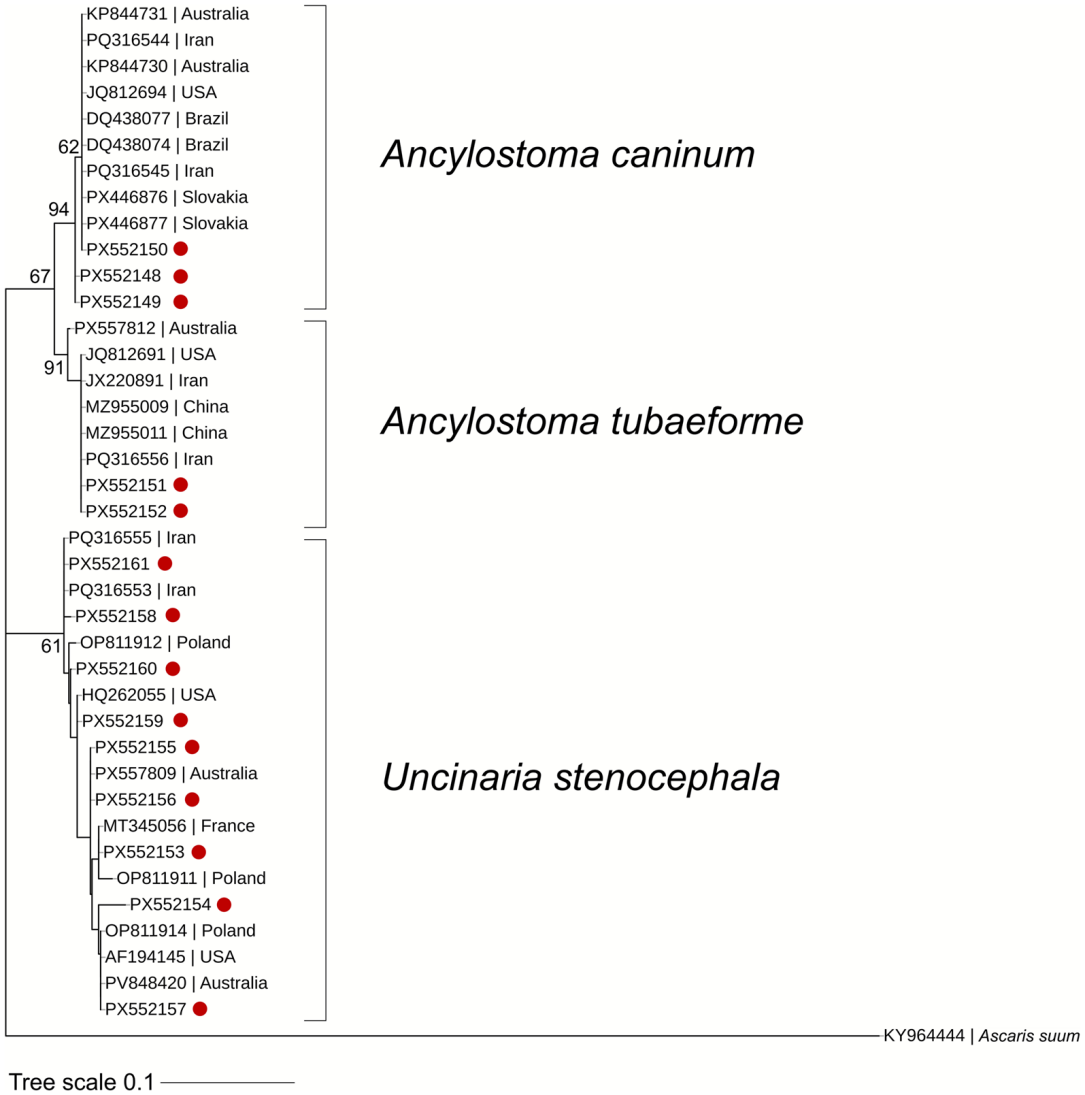
Bootstrap consensus tree, inferred from 1000 replicates, based on ITS-2 sequencing. The tree with the highest log likelihood (−769.44) is shown. The percentage of trees in which the associated taxa clustered together is shown next to the branches (values below 50% not shown). The tree is drawn to scale, with branch lengths measured in the number of substitutions per site. This analysis involved 40 nucleotide sequences: 14 obtained during the current study (marked by a red circle), 25 hookworm sequences downloaded from GenBank, and 1 sequence of *Ascaris suum* used as an outgroup. There were a total of 272 positions in the final dataset

All nematodes selected for molecular analyses were successfully sequenced, confirming the morphological identification of each hookworm species. Sequence similarity to representatives from GenBank ranged from 98.18% to 100% (Dataset 2), providing strong molecular support for the morphology-based diagnostic accuracy. For *U. stenocephala*, a total of nine haplotypes were identified, in *A. caninum* three haplotypes were detected, while *A. tubaeforme* had two haplotypes (Dataset 2). Their respective host associations and geographical distributions are shown in Figs. 6 and 7. The 14 unique sequences obtained were deposited in GenBank under accession numbers PX552148–PX552161. The sequence analysis showed global connectivity of hookworm populations (Fig. 8). *A. caninum* haplotypes from Romanian wild carnivores showed high similarity to isolates originating from Malaysia (dogs, humans) and Brazil. *A. tubaeforme* haplotypes were mostly similar with sequences from domestic cats in Iran. *U. stenocephala* exhibited the highest haplotype diversity, with close similarities recorded with specimens from France (wild boar), Alaska (arctic fox), and California (island foxes).

## Discussion

Hookworms (Ancylostomatidae) are globally recognized as important parasites owing to their medical and veterinary significance [2]. While their prevalence in domestic dogs and cats is well documented [13, 16, 28], the role of wild carnivores as reservoirs remains underexplored, especially in Europe [3]. The present study provides a comprehensive characterization of Ancylostomatidae in wild carnivores and is the first to molecularly identify *A. caninum*, *A. tubaeforme*, and *U. stenocephala* in Romania. Notably, we also report for the first time the Eurasian lynx as a host for *A. caninum*, expanding the known host range of this hookworm species.

The presence of *U. stenocephala*, *A. caninum*, and *A. tubaeforme* has been previously reported in European wild canids and felids in various studies, but such reports are focused almost exclusively on morphological identification.

*Uncinaria stenocephala* was found in six species of wild carnivores and reported in most countries across Europe, with an extensive geographic distribution, such as Austria [29], Belarus [30], Belgium [31], Croatia [32], Czech Republic [33], Denmark [34, 35], Estonia [36], Germany [37, 38], Hungary [37, 38], Ireland [39], Italy [40, 41], Latvia [42], Lithuania [43, 44], Poland [45, 46], Romania [9, 47], Serbia [48, 49], Slovakia [50], Slovenia [51], Spain [52], Sweden [53], Switzerland [54], Ukraine [55], and the UK [56].

*Ancylostoma caninum* has been reported in several wild carnivores species, namely *C. aureus*, *C. lupus*, *V. vulpes*, *N. procyonoides*, and *F. silvestris*, with records originating from different European countries such as Belarus [57], Denmark [34], Hungary [58], Italy [41], Latvia [42], Poland [59], Romania [48], Serbia [10, 48], Slovakia [50], Spain [60], and Ukraine [55].

*Ancylostoma tubaeforme* has been detected in different wild felid hosts, including *F. silvestris*, *L. lynx*, and *L. pardinus*, with reports originating from Croatia [61], Germany [62], Greece [63], Poland [64], Romania [11], Spain [65, 66], and Ukraine [55].

Recent studies from Germany [67] and Poland [4] morphologically and genetically identified hookworms in red foxes and raccoon dogs, as well as domestic dogs (*Canis familiaris*) and Eurasian badgers (*Meles meles*), but only from the genus *Uncinaria*. In Romania, prior studies documented hookworm infections in red foxes and wolves [9, 10], and *A. tubaeforme* in wildcats [11], but these reports were limited to morphological identification.

In Romania, the growing presence of wild animals in urban areas increases contact with both humans and domestic animals, creating risks for the spread of zoonotic diseases and raising concerns for conservation and public health [68]. Overall, the role of wildlife carnivores in the transmission of zoonotic hookworms, remains poorly understood owing to limited diagnostic tools and insufficient surveillance [3]. Our results highlight the possible role of the gray wolf, golden jackal, red fox, raccoon dog, European wildcat, and Eurasian lynx in the transmission of these zoonoses.

The genetic diversity observed across the hookworm species identified in this study reveals important patterns regarding host–parasite interactions and transmission dynamics among wild carnivores (Fig. 6). The greatest genetic diversity was detected in *U. stenocephala*, with nine different haplotypes. Interestingly, some of them were specifically associated with a single host (namely, UN1 and UN3 with gray wolves, UN2 with racoon dogs, and UN6–8 with golden jackals), while others were shared between multiple hosts (i.e., UN4 in gray wolves, red foxes, and golden jackals, and UN5 and UN9 in red foxes and golden jackals). One haplotype (AC1) of *A. caninum* was exclusively associated with golden jackals, the AC2 haplotype was shared by golden jackals and European wildcats, while AC3 was found in gray wolves and Eurasian lynx, confirming the overlapping habitats of the hosts. Both haplotypes of *A. tubaeforme*, a typically felid-associated species, was detected only in European wildcats.

The observed global connectivity of haplotypes highlights the potential for widespread circulation and exchange of hookworm lineages between wild and domestic hosts. The present results reinforce that lineages of *Ancylostoma* spp. infecting Romanian wildlife belong to globally distributed strains capable of infecting both animals and humans, probably transported by domestic dogs or humans. However, wildlife remains a suitable epidemiological marker for the evaluation of hookworm genetic diversity. The particularly high haplotype diversity observed for *U. stenocephala* is consistent with its wide ecological adaptability and evolutionary history across temperate and subarctic ecosystems, involving multiple carnivore hosts on different continents. Furthermore, the finding of different haplotypes within the same host is suggestive of multiple infection events. Such findings emphasize that wild carnivores in Europe not only sustain local transmission of hookworms but also fit into a broader, globally connected network of parasite circulation with potential veterinary and public health implications. While these findings are novel, their explanation could lie either in differences between host specificity or could be related to ecological interactions between hosts owing to shared habitats. Moreover, certain findings may be related to low sample sizes. Certainly, more studies are required to understand the possible links between haplotypes and hosts, and the genetic characterization of hookworms should also include domestic hosts.

This study has several limitations that should be acknowledged. In some host individuals, only a limited number of nematodes were recovered, while in others the specimens had been stored for a long time, potentially affecting their integrity and suitability for analysis. Moreover, the geographical distribution of sampled hosts across Romania was uneven, which may limit the representativeness of the results.

## Conclusions

Overall, the findings of the present study highlight the significant role of postmortem examination in wild carnivores and the value of combining morphological and molecular confirmation methods for accurate results. This integrated approach allowed the detection of new host–parasite associations and provides new insights into the ecology of hookworms in European wild carnivores, which is essential for increasing general knowledge on this topic. However, further studies are required to achieve a comprehensive understanding of the distribution and transmission dynamics of these parasites, health implications, and zoonotic risks.

## Supplementary Information

Supplementary material 1. Dataset 1. Collected samples dataset. Each collected sample is characterized by a set of specific attributes: Code (a unique identifier assigned to each sample), Host (the animal species from which the sample was collected), Date of collection (the date when the sample was collected), Date of examination (the date when the necropsy examination was done), Sex (sex of the host species), Age (the category of animal; e.g., juvenile, adult), Locality (the geographical location where the animal was found or collected), Latitude and Longitude (the exact geographical coordinates of the collection site), Digestive hookworms (refers to the presence or absence of the hookworms).

Supplementary material 2. Dataset 2. Sequence data of hookworm isolates, including accession numbers, sequencing IDs, host information, and similarity (%) with reference species.

## Data Availability

All data generated or analyzed are provided within the manuscript and in the Supplementary Information files.
